# An Improved Rapidly-Exploring Random Trees Algorithm Combining Parent Point Priority Determination Strategy and Real-Time Optimization Strategy for Path Planning

**DOI:** 10.3390/s21206907

**Published:** 2021-10-18

**Authors:** Lijing Tian, Zhizhuo Zhang, Change Zheng, Ye Tian, Yuchen Zhao, Zhongyu Wang, Yihan Qin

**Affiliations:** School of Technology, Beijing Forestry University, Beijing 100083, China; T7190221@bjfu.edu.cn (L.T.); zhangzhizhuo@bjfu.edu.cn (Z.Z.); tytoemail@bjfu.edu.cn (Y.T.); zhaoyuchen@bjfu.edu.cn (Y.Z.); wangzhongyu@bjfu.edu.cn (Z.W.); linxinyi031@bjfu.edu.cn (Y.Q.)

**Keywords:** rapidly-exploring random trees, manipulator, priority determination, real-time optimization, path planning

## Abstract

In order to solve the problems of long path planning time and large number of redundant points in the rapidly-exploring random trees algorithm, this paper proposed an improved algorithm based on the parent point priority determination strategy and the real-time optimization strategy to optimize the rapidly-exploring random trees algorithm. First, in order to shorten the path-planning time, the parent point is determined before generating a new point, which eliminates the complicated process of traversing the random tree to search the parent point when generating a new point. Second, a real-time optimization strategy is combined, whose core idea is to compare the distance of a new point, its parent point, and two ancestor points to the target point when a new point is generated, choosing the new point that is helpful for the growth of the random tree to reduce the number of redundant points. Simulation results of 3-dimensional path planning showed that the success rate of the proposed algorithm, which combines the strategy of parent point priority determination and the strategy of real-time optimization, was close to 100%. Compared with the rapidly-exploring random trees algorithm, the number of points was reduced by more than 93.25%, the path planning time was reduced by more than 91.49%, and the path length was reduced by more than 7.88%. The IRB1410 manipulator was used to build a test platform in a laboratory environment. The path obtained by the proposed algorithm enables the manipulator to safely avoid obstacles to reach the target point. The conclusion can be made that the proposed strategy has a better performance on optimizing the success rate, the number of points, the planning time, and the path length.

## 1. Introduction

Whether for mobile robots such as AGV (automated guided vehicle) carts working in automated workshops, or robotic arms such as agricultural manipulators in the field, the core of automation for autonomous robots is path planning. Path planning is the process of finding an obstacle-free path from an initial position to a target position in a known or partially known environment [1].

Path planning is one of the most important research focuses of robots. Chinthaka Premachandra et al. completed the robot’s path planning in an indoor environment by a self-localization method through baseboard recognition and image processing [2]. Wenzhou Chen et al. used distributed sonar sensors to calculate the distance between the receiver and the generator in real time to control the moving path of the robot [3]. Chinthaka Premachandra et al. proposed a hybrid aerial-terrestrial robot system to help UAVs avoid obstacles during the movement [4]. Path planning algorithms can usually be divided into three types. The first type is the bionic-based path planning algorithm [5], of which the ant colony algorithm is a common one and has the advantages of robustness and environmental adaptability, but its convergence speed is slow and very easy to fall into the local optimum. The second type is the map-based path planning algorithm, of which the A* algorithm (Optimal A-algorithm), with the optimal surrogate and prognostic functions, is a commonly used one and has the advantages of heuristic search and the obtained path is optimal, but its planning time is long and not applicable to high-dimensional space [6]. The third type is the sampling-based path planning algorithm [7], of which the most commonly used one is the RRT (rapidly-exploring random trees) algorithm [8]. As an efficient path-planning method in a multi-dimensional space, the RRT algorithm uses an initial point as the root point and generates a random extended tree by randomly sampling and adding leaf points. When a leaf point in the random tree contains a target point or enters a target region, a path from the initial point to the target point, consisting of tree points, can be found in the random tree. The RRT algorithm is the most popular path planning algorithm due to the rapidness, probabilistic completeness, and good scalability [9,10,11,12,13].

However, the RRT algorithm also has many disadvantages. Among the main disadvantages of the RRT algorithm, one is that the whole random tree needs to be traversed to search the parent point in the process of new point generation, which consumes a lot of computation time. The other is the large number of redundant points generated during the path generation process. To address the above problems, this paper proposes an improved RRT algorithm based on the PPD strategy (the strategy of parent point priority determination) to speed up the path planning, and further optimizes the efficiency of the algorithm by incorporating the RO strategy (real-time optimization) on this basis. The PPD strategy would shorten the path planning time and the RO strategy would reduce the number of redundant points. Finally, MATLAB-based three-dimensional comparison simulation experiments were conducted, and the experimental results showed that the proposed algorithm has a faster planning speed and can generate fewer redundant points, which has a better performance compared with other improved algorithms.

The rest of this paper is organized as follows. Section 2 introduces the related work and Section 3 presents the proposed RRT algorithm in three parts including the RRT algorithm, the PPD strategy, and the RO strategy. Section 4 shows the simulation results of the proposed algorithm in three-dimensional space and the experiments using IRB1410 in the laboratory. In the following, a discussion is presented in Section 5. Finally, Section 6 presents the conclusions of this paper.

## 2. Related Work

The RRT algorithm has been widely used in the field of robot motion and path planning. However, the paths obtained are not optimal, mainly because of several aspects such as path planning time, the number of points, and the path length. To solve these shortcomings, many improved algorithms based on the RRT algorithm have been proposed to promote the path-planning efficiency. LaValle and Kuffner proposed a bidirectional extended random tree algorithm [14] that generated two random trees from the starting point and the target point simultaneously, expanding them in space separately. This algorithm used a greedy strategy to reduce the number of iterations in the path generation process. Sertac and Emilio proposed an asymptotically optimal RRT* algorithm (an improved algorithm for progressively optimizing path length by reselecting parent point) [15], which changed the selection of the parent point and used a cost function to select the point with the smallest cost in the neighborhood of the extended point as the parent point, thus reducing the cost of path generation and improving the search efficiency. Jordan et al. borrowed the RRT-Connect (bidirectional extended random tree) algorithm idea and proposed a bidirectional extended RRT* algorithm, namely B-RRT* (bidirectional version of RRT*) algorithm [16]. Wang Kun et al. proposed a two-way extended RRT* algorithm for heuristic search, which reduced the number of iterations to a certain extent [17]. Jordan proposed the B-RRT* algorithm [18], which used the strategy of reselecting the parent point and rewiring two trees to speed up the algorithm convergence. Qureshi et al. added the heuristic strategy to the B-RRT* algorithm and proposed the IB-RRT* (Intelligent bidirectional-RRT*) algorithm [16]. Qureshi et al. combined the artificial potential field method with the RRT* algorithm to improve the convergence speed of the algorithm [19]. Barfoot proposed the Inform-RRT* algorithm [20] to narrow the search range and speed up the convergence of the algorithm on the basis of obtaining feasible paths. Mashayekhi et al. proposed the Informed-RRT*-Connect algorithm [21], which used a bidirectional tree to quickly find the initial path before using a subset of heuristics to directly sample to accelerate convergence, and the heuristic algorithm performed better in the improved RRT*-Connect algorithm. While the RRT* algorithm and its improved algorithm helped to reduce the path length, their planning times were several times longer than that of the RRT algorithm. Although the RRT-Connect algorithm and its improved algorithm had a slight reduction in the number of redundant points and planning time, the optimization effect was not significant and the path length was much longer than that of the RRT algorithm.

The above improvement algorithms have different advantages. In this paper, we focused on both the path planning time and the number of redundant points. Two different improved strategies for each of the two aspects combined are proposed.

## 3. Methods

### 3.1. The Rapidly-Exploring Random Tree Algorithm (RRT)

The rapidly-exploring random tree algorithm is a probability-complete global path planning algorithm that obtains path points by random sampling in the search space and then achieving a feasible path from the start point to the goal point. The specific process is shown in Algorithm 1.
**Algorithm 1.** RRT algorithm.a. Initialize the random tree $P_{init}$.b. Select a random point $P_{rand}$ in the search space.c. Traverse the random tree and find the closest point to $P_{rand}$ in the random tree, named $P_{parent}$.d. Intercept the step length ρ along the direction from $P_{near}$ to $P_{rand}$ to get a new point $P_{new}$.e. Repeat the above steps b–d until the target point $P_{goal}\;$ is added to the random tree.

**Figure 1:**
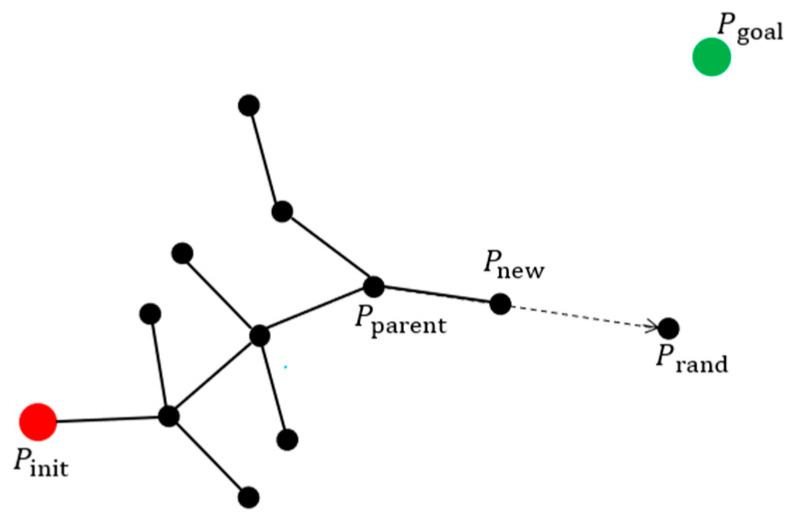
Random tree expansion diagram for the RRT algorithm. The red circle indicates the starting point and the green circle indicates the target point. Black circle indicates the path point, black solid line indicates the path, and black dashed line with arrow indicates the current expansion direction of the random tree. $P_{rand}$ denotes the randomly sampled point, $P_{parent}$ denotes the parent point, and $P_{new}$ denotes the new point.

The random tree expansion diagram for the RRT algorithm is shown in Figure 1. The RRT algorithm generates new points by random sampling in the workspace. In the random tree expansion process, searching $P_{parent}$ requires traversing the entire random tree, a process that takes a lot of time when the random tree grows relatively large, which in turn leads to a slow path-planning speed of the algorithm. The sampling method of the RRT algorithm is highly random, which results in a large number of redundant points. For these problems, two improved strategies are proposed in this paper.

### 3.2. The Strategy of Parent Point Priority Determination (PPD)

In order to save time in traversing the whole random tree in the process of determining the parent point, this paper proposes a strategy of parent point priority determination to simplify this process and thus shorten the path planning time. The strategy of parent point priority determination is an improved strategy based on the RRT algorithm, whose core idea is to prioritize the parent point of the next new point before random sampling. Compared with the RRT algorithm, the improved algorithm based on the PPD strategy saves time in finding the $P_{parent}$ and therefore speeds up the execution of the algorithm. The specific process is shown in Algorithm 2, where $D_{new}$ and $D_{parent}$ denote the distance from the new point to the target point and the distance from the parent point to the target point, respectively, which can be calculated by Equation (1).

(1) $$\{\begin{array}{c} D_{new}=\sqrt{{\left(P_{new\left(x\right)}-P_{goal\left(x\right)}\right)}^{2}+{\left(P_{new\left(y\right)}-P_{goal\left(y\right)}\right)}^{2}}\\ D_{parent}=\sqrt{{\left(P_{parent\left(x\right)}-P_{goal\left(x\right)}\right)}^{2}+{\left(P_{parent\left(y\right)}-P_{goal\left(y\right)}\right)}^{2}} \end{array}$$

**Algorithm 2.** PPD-RRT algorithm.a. Initialize the random tree $P_{init}$.b. Set the point $P_{init}$ as the parent point $P_{parent}$ of the next expansion.c. Get four random points $P_{rand1}$$\sim P_{rand4}$ on the circumference of the circle with the parent point $P_{parent}$ as the center and the step length ρ as the radius.d. Select the closest point to the target point in $P_{rand1}$$\sim P_{rand4}$ as the random point $P_{rand}$.e. Connect parent point $P_{parent}$ to the random point $P_{rand}$, the random point $P_{rand}$ is the new point $P_{new}$.f. Use Equation (1) to calculate $D_{new}$ and $D_{parent}$ respectively, and choose the one which is closer to the target point as the parent point $P_{parent}$ for the next expansion.g. Repeat the above steps c–f until the target point $P_{goal}$ is added to the random tree.

**Figure 2:**
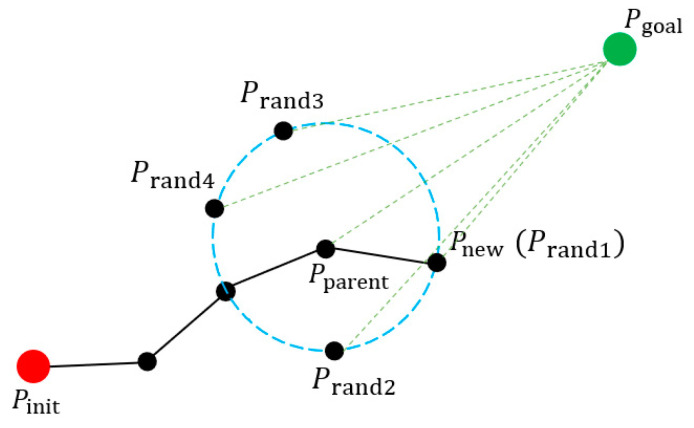
Random tree expansion diagram of the improved algorithm based on the PPD strategy. The red circle indicates the starting point, and the green circle indicates the target point. The black circle indicates the path point, the solid black line indicates the path. and the green dashed line indicates the distance from the point to the target point. The blue dashed line indicates the circumference of the circle with the parent point as the center and the step length ρ as the radius, which is the random sampling space. $P_{rand1}$~$P_{rand4}$ denote random sampling points, $P_{parent}$ denotes parent point, and $P_{new}$ denotes the new point.

The random tree expansion diagram of the improved algorithm based on the PPD strategy is shown in Figure 2. In the process of generating new points, as the random tree becomes larger and there are more and more points in the random tree, traversing the random tree to search the parent point consumes a lot of computational time. In this article, the parent point is determined before the new point is generated, which can greatly save the path-planning time, and the larger the random tree gets, the more obvious this effect becomes.

### 3.3. The Strategy of Real-Time Optimization (RO)

The improved algorithm based on the PPD strategy can greatly reduce the path planning time, but the number of path points still needs to be optimized. Therefore, a strategy of real-time optimization was proposed in this paper. We calculated the distance $D_{new}$ between the new point and the target point, and if $D_{new}$ can satisfy both Equations (2) and (3), the generation of the new point is considered to beneficial to the growth of the random tree. The core idea of the real-time optimization strategy is to determine whether the new point has a positive impact on the subsequent growth of the random tree immediately after the new point is generated, as shown in the process of Algorithm 3. The real-time optimization of the RO policy can effectively reduce the number of redundant points of the random tree.
**Algorithm 3.** PPRO-RRT algorithm.a. Initialize the random tree $P_{init}$.b. Set the point $P_{new}$ by the exploration process of PPD-RRT algorithm.c. Calculate the distance from the new point, the parent point and its two nearest ancestor points to the target point according to Equation (2), respectively.d. Determine whether the new point $P_{new}$ is reserved according to Equation (3), and if it is satisfied, then it is reserved.e. Repeat the above steps b–d until the target point $P_{goal}$ is added to the random tree.

(2) $$\{\begin{array}{c} D_{ancestor1,2}=\sqrt{{\left(P_{ancestor1,2\left(x\right)}-P_{goal\left(x\right)}\right)}^{2}+{\left(P_{ancestor1,2\left(y\right)}-P_{goal\left(y\right)}\right)}^{2}}\\ D_{parent}=\sqrt{{\left(P_{parent\left(x\right)}-P_{goal\left(x\right)}\right)}^{2}+{\left(P_{parent\left(y\right)}-P_{goal\left(y\right)}\right)}^{2}} \end{array}$$

(3) $$\left(D_{ancestor1}>D_{new})\;or\;(D_{ancestor2}>D_{new})\;or\;(D_{parent}>D_{new}\right)$$

**Figure 3:**
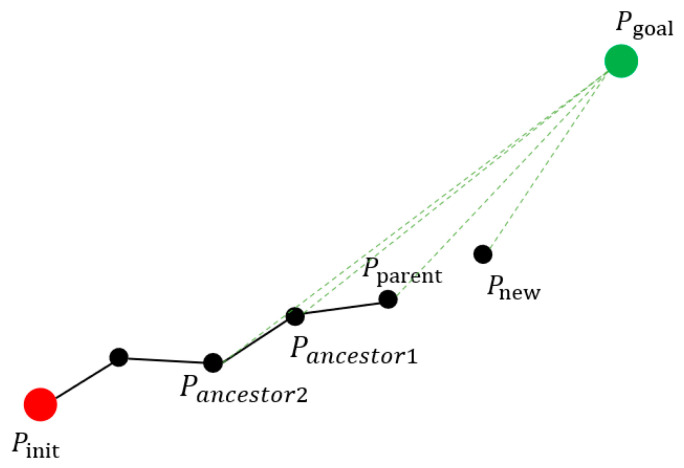
Random tree expansion diagram of the improved algorithm based on the RO strategy. The red circle indicates the starting point, and the green circle indicates the target point. The black circle indicates the path point, the solid black line indicates the path, and the green dashed line indicates the distance from the point to the target point. $P_{ancestor1}$~$P_{ancestor2}$ denote the ancestor points, $P_{parent}$ denotes the parent point, and $P_{new}$ denotes the new point.

The random tree expansion diagram of the improved algorithm based on the RO strategy is shown in Figure 3. Once a new point is generated, Equations (2) and (3) are used to decide the point to be left. If this new point does not contribute to the growth of the random tree, it is rejected, which avoids growing more redundant points from that point. Therefore, the number of redundant points is greatly reduced by a real-time judgment that prevents the generation of more redundant points.

## 4. Experiment and Analysis

The PPD strategy can effectively reduce the path planning time, and the RO strategy can reduce the number of redundant points. In order to further evaluate the performance of the PPD-RRT algorithm and the PPRO-RRT (parent point priority determination-real-time optimization-RRT) algorithm, we will conduct simulation experiments in 3-dimensional space for the above improved algorithm and the existing improved algorithm to verify the high-dimensional reliability and efficiency of the improved algorithm in this paper.

### 4.1. Simulation Experiments

**Figure 4:**
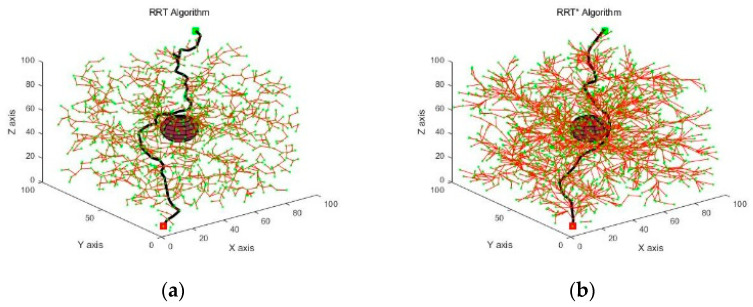

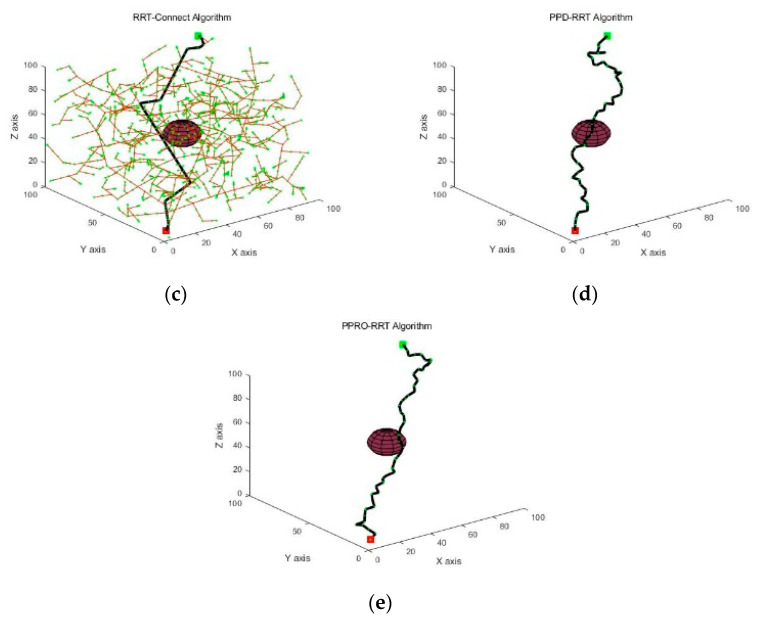
Paths planned by various algorithms in a simple environment. The red cube indicates the starting position and the green cube indicates the target position. The red sphere indicates the obstacles in the environment, the green dot indicates the points obtained by sampling during the path planning process, the red solid line indicates the branches of the random tree, and the black solid line indicates the feasible paths obtained. (**a**) RRT algorithm; (**b**) RRT* algorithm; (**c**) RRT-Connect algorithm; (**d**) PPD-RRT algorithm; (**e**) PPRO-RRT algorithm.

**Figure 5:**
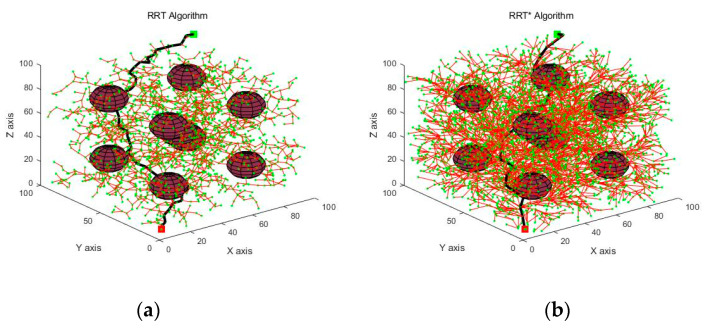

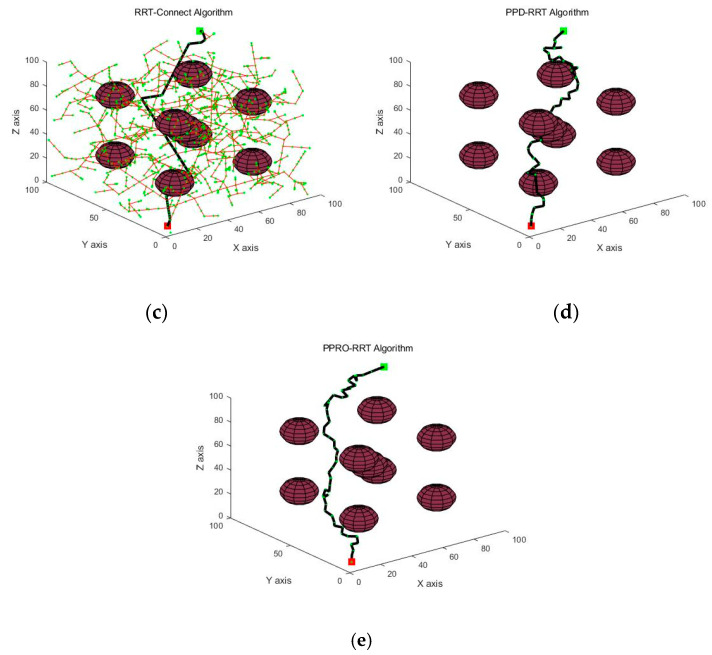
Paths planned by various algorithms in a complex environment. The red cube indicates the starting position and the green cube indicates the target position. The red sphere indicates the obstacles in the environment, the green dot indicates the points obtained by sampling during the path planning process, the red solid line indicates the branches of the random tree, and the black solid line indicates the feasible paths obtained. (**a**) RRT algorithm; (**b**) RRT* algorithm; (**c**) RRT-Connect algorithm; (**d**) PPD-RRT algorithm; (**e**) PPRO-RRT algorithm.

The simulation experimental platform was configured with MATLAB 2019b, 64-bit Windows 10, processor Inter(R) Core (TM) i7-10700F CPU @ 2.90 GHz, and 16 GB of memory. The experimental simulation area was a 100 × 100 × 100 cube area with the starting position [5, 5, 5] and the target position [95, 95, 95]. The experimental environment was designated as a simple and complex one according to the number of obstacles [22]. Figure 4 and Figure 5 show the simulation results of various algorithms in simple and complex environments.

The simulation results in Figure 4 and Figure 5 showed that there was a significant reduction in the number of sampling points because the PPRO-RRT algorithm incorporated the idea of PPD and RO.

### 4.2. Results and Analysis

In order to avoid the influence of the randomness of the path planning algorithm on the experimental results, each method was tested for 5000 iterations in the same environment, and the upper limit of the number of single iterations was set to 1000. The proposed algorithm was compared with other algorithms in terms of the success rate, the number of path points, the path planning time, and the path length. The results of each experiment are shown in Table 1, Table 2, Table 3 and Table 4.

Table 1 indicates the success rate of various path planning algorithms in two kinds of experimental environments. The experimental results showed that the success rates of the PPD-RRT and PPRO-RRT path planning algorithms were significantly higher than those of the other algorithms. The success rates of the PPD-RRT and PPRO-RRT algorithms were 99.80% and 100.00% in simple environments and 99.48% and 99.99% in complex environments, respectively. The success rate improvement was significant compared with other algorithms. The PPRO-RRT algorithm achieved success rate increases of 94.88% and 96.05% compared with the RRT algorithm; 94.32% and 95.37% compared with the RRT* algorithm; and 50.30% and 48.11% compared with the RRT-Connect algorithm. With a set number of iterations of 1000, the PPRO-RRT algorithm greatly enhances the success rate of the path planning algorithm.

**Table 2:** The number of random tree points of various algorithms in two kinds of experimental environments.

	RRT	RRT*	RRT-Connect	PPD-RRT	PPRO-RRT
Simple environments	831	829	446	153	56
Complex environments	834	823	447	152	55

Table 2 indicates the number of random tree points obtained by various path planning algorithms. For each of the algorithms, the number of random tree points hardly varied with the complexity of the environment. Compared with the RRT algorithm, the number of random tree points of the PPD-RRT algorithm was reduced by approximately 81.59% and 81.76% in two kinds of experimental environments, and after adding the RO idea, the number of points was reduced by about 93.25% and 93.32%. Compared to the RRT* and RRT-Connect algorithms, the number of random tree points of the PPRO-RRT algorithm was reduced by 93.23% and 87.42% in the simple environments and 93.23% and 87.55% in the complex environments, respectively.

**Table 3:** The path planning time(s) of various algorithms in two kinds of experimental environments.

	RRT	RRT*	RRT-Connect	PPD-RRT	PPRO-RRT
Simple environments	0.0240	0.0704	0.0318	0.0024	0.0017
Complex environments	0.0388	0.1318	0.0461	0.0044	0.0033

Table 3 indicates the path planning time of different algorithms in two kinds of experimental environments. For each of the proposed algorithms, the path planning time was approximately reduced by one order of magnitude. In the simple experimental environment, the path planning time of the PPD-RRT and PPRO-RRT algorithms was 90% and 92.92% shorter than that of the RRT algorithm, respectively. In the complex experimental environment, the path planning time of the PPD-RRT and PPRO-RRT algorithms was 88.66% and 91.49% shorter than that consumed by the RRT algorithm, respectively. As the RRT* algorithm and the RRT-Connect algorithm both require a longer path planning time than the RRT algorithm, the PPD-RRT algorithm and the PPRO-RRT algorithm were far superior to the RRT* algorithm and the RRT-Connect algorithm in terms of path planning time. Compared with the RRT* and RRT-Connect algorithms, the path planning time of the PPRO-RRT algorithm was reduced by 97.59% and 94.65% in the simple environments and 97.05% and 92.84% in the complex environments, respectively.

**Table 4:** The path length [23] of various algorithms in two kinds of experimental environments.

	RRT	RRT*	RRT-Connect	PPD-RRT	PPRO-RRT
Simple environments	221.42	190.15	231.34	241.95	203.96
Complex environments	225.00	195.22	240.49	237.67	202.48

Table 4 indicates the path length of the various algorithms in the two kinds of experimental environments. Compared with the RRT algorithm, the path length of the PPD-RRT algorithm became longer in two kinds of experimental environments due to the restricted sampling area. After adding the RO idea, the path length of the PPRO-RRT algorithm was reduced by 7.89% and 10.01% in simple environments and complex environments. While the path length of the PPRO-RRT algorithm was longer than that of the RRT* algorithm, the difference was not significant and was far superior to the RRT* algorithm in other aspects. Compared with the RRT-Connect algorithm, the path length of the PPRO-RRT algorithm was reduced by 11.84% and 15.81% in simple environments and complex environments.

From the above analysis, the PPRO-RRT algorithm can converge at a small number of iterations, and the success rate reaches close to 100% with a set upper limit of 1000 iterations. The PPRO-RRT algorithm saves the process of searching parent points, which greatly saves the path planning time; the real-time judgment of whether new points should be retained reduces the generation of more redundant points. We can conclude that the PPRO-RRT algorithm has significant advantages over the RRT algorithm in terms of success rate, number of points, planning time, and path length.

### 4.3. Experiments on the Manipulator

**Figure 6:**
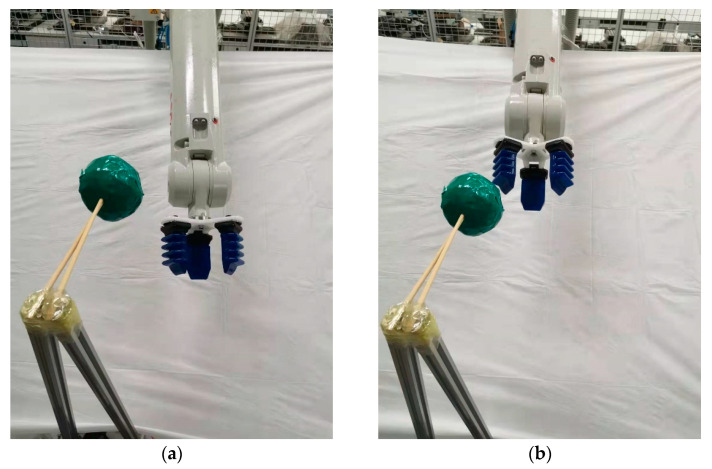

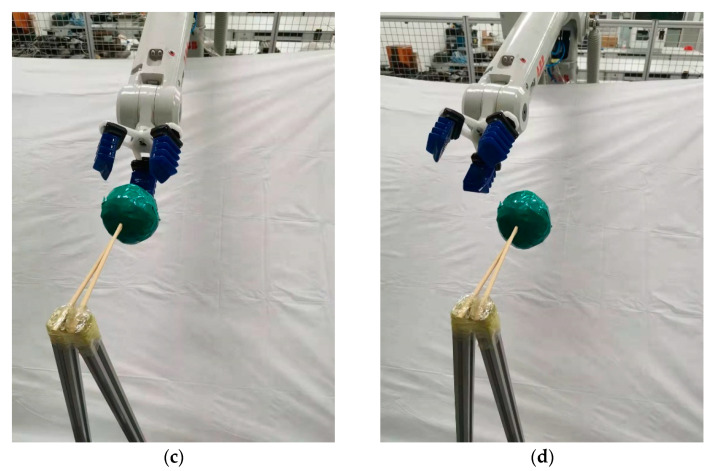
Path sequence with the PPRO-RRT algorithm in obstacle avoidance. (**a**–**d**) represent different states of the manipulator and obstacle at each moment.

To demonstrate the feasibility and effectiveness of the algorithm proposed in this paper, an experimental platform for path planning was built in a laboratory environment using the IRB1410 robot arm. A random position (1.289 m, 0.013 m, 0.873 m) was selected as the position of an obstacle in the manipulator workspace. In this scene, the path point of the manipulator end-effector is obtained by the PPRO-RRT algorithm, and then the manipulator is controlled by the program procedure from the starting point (1.241 m, 0.156 m, 0.703 m) around the obstacle to the target point (1.166 m, −0.087 m, 0.921 m), and the sequence of the path is shown in Figure 6.

**Figure 7:**
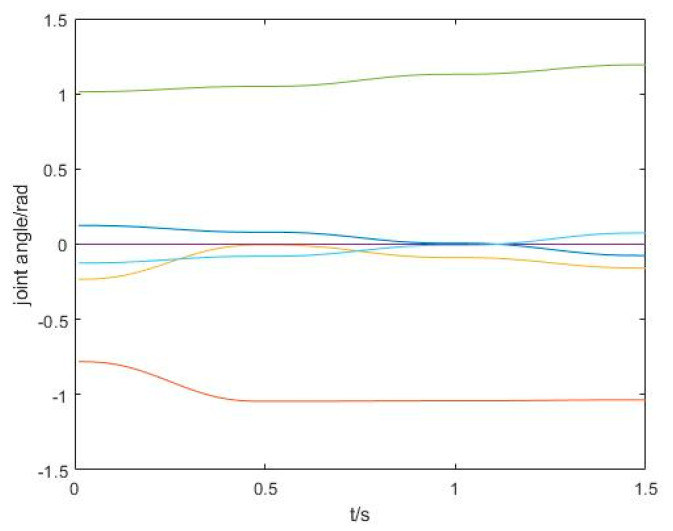
Variation of each joint angle during the process of obstacle avoidance.

In this project, the individual joint angles of the robot arm change, as shown in Figure 7, from which we can see that the individual joint angles change smoothly.

## 5. Discussion

In this paper, simple and complex environments were chosen to verify the feasibility of the proposed algorithm. The experimental results showed that the PPRO-RRT algorithm had better performance in both environments than the RRT algorithm, the RRT* algorithm, and the RRT-Connect algorithm. However, in the field conditions, the position of the obstacles may change or there may be narrow gaps between the obstacles. The algorithm in this paper does not fit in the above scene, which creates some limitations on the path planning of the manipulator. Therefore, the following research will focus on enhancing the applicability of the algorithm to a wider range of scene.

In addition, in the real environment, the manipulator may collide with the obstacle, which is because the actual size of the manipulator needs to be considered during the experiment in the real scene, which would make the collision detection algorithm more complicated and increase the running time of the algorithm. In order to simplify the collision detection algorithm, the links of the manipulator are abstracted as lines and the obstacle is inflated. The actual size of the manipulator is added to the obstacle so that the complex process of collision detection is transformed into a problem of the position relationship between a line and a sphere. Experimental results showed that with the proposed algorithm in this paper, the manipulator could safely reach the target point from the starting point.

## 6. Conclusions

In order to simplify the complex process of determining the parent point that needs to traverse the random tree and reduce the number of redundant points in the random tree, a PPRO-RRT algorithm, combined the PPD strategy with the RO strategy, was proposed in this paper. The following conclusions were drawn through experiments and comparative analysis: (1) the PPD strategy significantly speeds up path planning, and the RO strategy reduces the number of redundant points; (2) the PPRO-RRT algorithm outperforms the other improved RRT algorithms for both simple and complex environments; (3) the experimental results by IRB1410 shows that the manipulator can safely avoid obstacles using the PPRO-RRT algorithm. However, compared with the RRT* algorithm, the algorithm proposed in this paper may produce longer paths and would be modified in the future in terms of shorter path lengths. The improved algorithm in this paper is relatively unsuitable for the scene with narrow channels. In terms of practical applications, the improved algorithm in this paper can be applied to the path planning problems of intelligent vehicles, manipulators, UAVs, etc. to speed up their path planning.

## Figures and Tables

**Table 1:** The success rate of various algorithms in two kinds of experimental environments.

	RRT	RRT*	RRT-Connect	PPD-RRT	PPRO-RRT
Simple environments	5.12%	5.68%	49.70%	99.80%	100.00%
Complex environments	3.94%	4.62%	51.88%	99.48%	99.99%
